# Supplementary material for: Towards high resolution, validated and open global wind power assessments
Source: Nat Commun. 2026 Jan 14;17:539. doi: 10.1038/s41467-026-68337-z (PMC12804690; doi:10.1038/s41467-026-68337-z)
Supplement: Supplementary file 1 — Supplementary Information [file 41467_2026_68337_MOESM1_ESM.pdf]

Supplementary Information

Towards high resolution, validated and open global wind power assessments

E. U. Peña-Sánchez[1,2,†], P. Dunkel[1,2, †,*], C. Winkler[1,2,†], H. Heinrichs[1], F. Prinz[1], J.M. Weinand[1], R. Maier[1,2], S. Dickler[1], S. Chen[1,3], K.Gruber[4] , T.Klütz[1], J. Linßen[1], and D. Stolten[1,2]

1 Forschungszentrum Jülich GmbH, Institute of Climate and Energy Systems, Juelich Systems Analysis, 52425 Jülich, Germany

2 RWTH Aachen University, Chair for Fuel Cells, Faculty of Mechanical Engineering, 52062 Aachen, Germany

3 Forschungszentrum Jülich GmbH, Institute of Bio- and Geosciences - Agrosphere (IBG-3), 52425 Jülich, Germany

4 Institute for Sustainable Economic Development, University of Natural Resources and Life Sciences, Vienna, Austria

† These authors contributed equally to this work
*corresponding author: p.dunkel@fz-juelich.de

1. Supplementary Material

1.1 Supplementary tables and figures

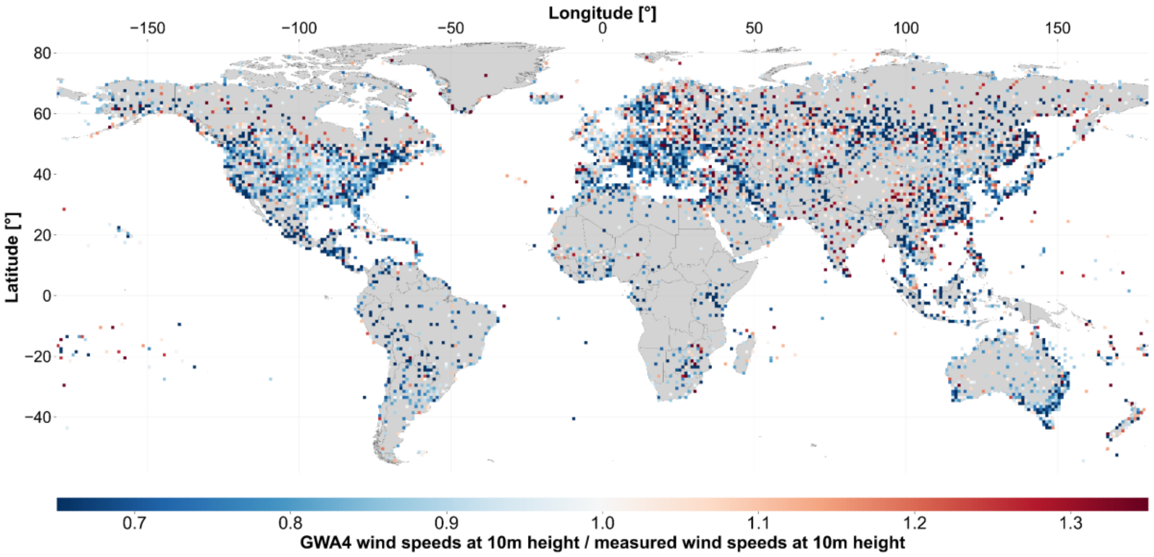

Supplementary Figure 1: Comparison of GWA4 and measured wind speeds at 10 m height. Global map showing the ratio between wind speeds (ws) from the Global Wind Atlas 4 (GWA4) and time-averaged measured wind speeds at 10 m height. Measurements sourced from [19,20]. The ratio is calculated at each measurement location as $ws_{GWA4}/ws_{measurement}$ and aggregated to a regular raster by averaging all values within each raster cell. Blue shading represents locations with underestimation of GWA4, and red shading overestimation. Country shapes from GADM [3]. Source data are provided as a Source Data file.

Supplementary Table 1: Aggregated mean error by source and location

Turbine type	Source - Location	Mean error (absolute)	Mean error (relative)	Data class
**Offshore**	**Energy Numbers - North Atlantic Ocean**	-0.018	-0.038	Aggregated
	**Fraunhofer - Baltic Sea**	0.013	0.028	hourly resolved
	**Fraunhofer - North Atlantic Ocean**	0.065	0.181	hourly resolved
**Onshore**	**DEA - Denmark**	-0.004	0.001	hourly resolved
	**Denker And Wulf - Germany**	0.082	0.313	hourly resolved
	**EMI – New Zealand**	0.064	0.167	hourly resolved
	**NVE - Norway**	0.039	0.108	hourly resolved
	**Plan- Og Landdistriktsstyrelsen - Denmark**	0	0.013	Aggregated
	**The U.S. Wind Turbine Database - USA**	0.003	0.04	Aggregated
	**UEPS - Brazil**	-0.179	-0.431	hourly resolved

1.2 Wind speed measurement data

As the data formatting, level of detail and temporal resolution varies among the data sources, the meteorological data was standardized (UTC time convention, averaged one-hour resolution) using the xarray python package [1]. Afterward, the standardized data was combined into one data set. For the meteorological data, most times quality control information provided by the data source was utilized to filter out erroneous measurements (e.g. no valid recording, negatives, duplicated values, etc.). In cases where tall masts had multiple instruments at the same height, due to the wind shadow effect, the measurement with the higher value was consistently selected.

Additionally, for the validation, wind speeds outside below 3 m/s were filtered out as they are not relevant for wind power generation due to the operational range of wind turbines.

Afterwards, the measurements were saved into one netCDF file per location and measurement height.

Although the wind speed in ERA5 is stated as instantaneous, it is necessary to time-average the wind speed measurements to approximate the characteristics of the reanalysis data. While the reanalysis data provides an average over an area defined by the model's grid spacing, the measurement is a point measurement that is subject to significant local fluctuations in wind speed. Therefore, the optimal averaging period for the wind speed measurement data was investigated. The wind speed measurements averaged over 10, 20, 40, and 60-minute periods were compared to the corresponding ERA5 values using metrics such as the Pearson correlation coefficient, root mean square error (RMSE), mean absolute error (MAE), and mean deviation and are shown in Supplementary Figure 2, Supplementary Figure 3, Supplementary Figure 4 and Supplementary Figure 5.

Nearly all metrics exhibit consistent improvement as the averaging period increases from 10 min to 60 minutes. Even higher measurement periods further improve the metrics. Nevertheless, to uphold the one-hour temporal resolution of ERA5 data, a one-hour averaging period was selected, as the benefits of longer averaging periods fail to outweigh the loss in temporal resolution.

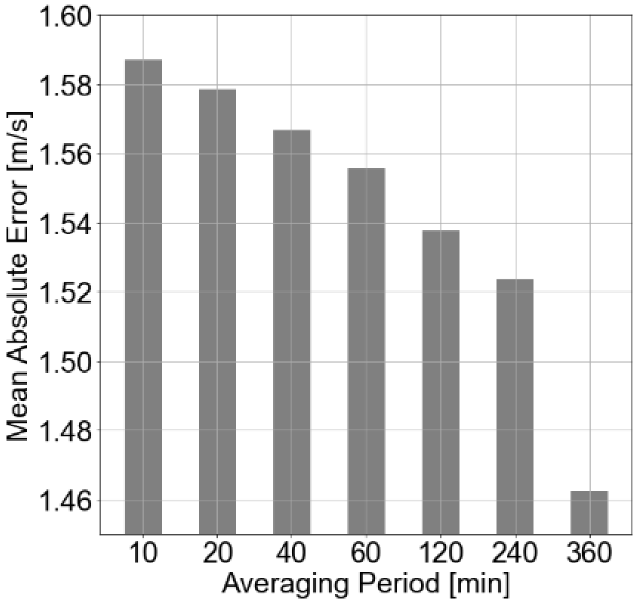

Supplementary Figure 2: Mean Absolute Error for different averaging periods. Bar chart showing the mean absolute error (MAE) between measured wind speeds and corresponding ERA5 values as a function of the temporal averaging period. Each bar represents the MAE calculated for a specific averaging interval.

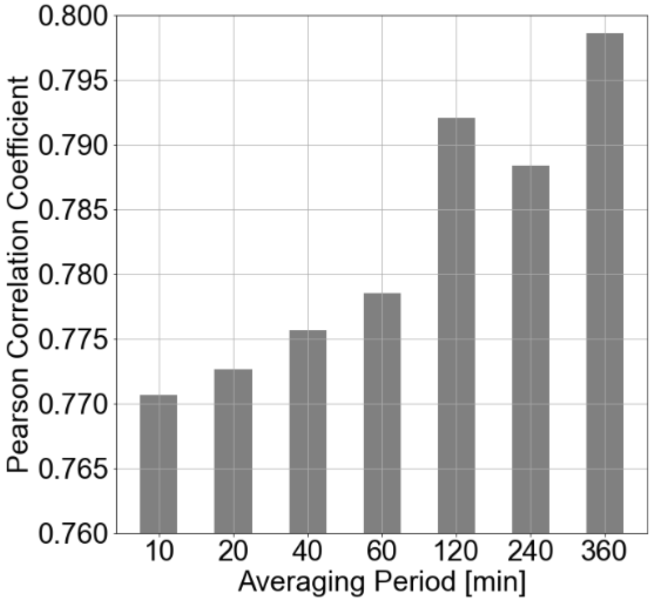

Supplementary Figure 3: Pearson correlation coefficient for different averaging periods. Bar chart showing the Pearson Correlation Coefficient between measured wind speeds and corresponding ERA5 values as a function of the temporal averaging period. Each bar represents the Pearson Correlation Coefficient calculated for a specific averaging interval.

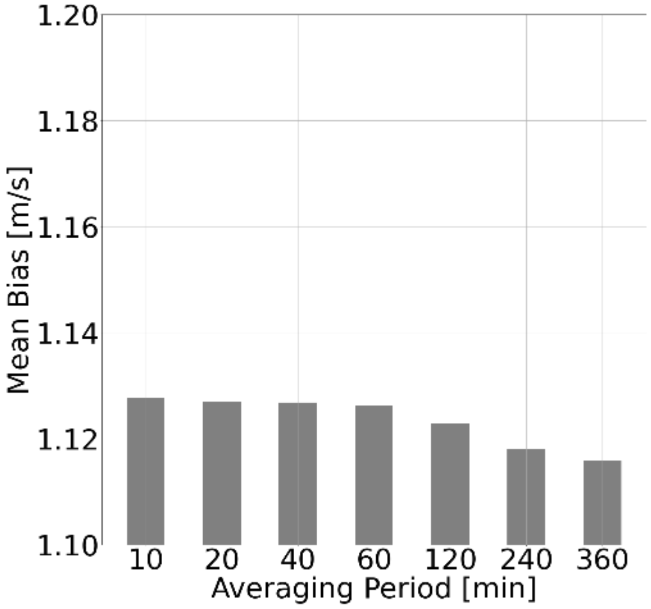

Supplementary Figure 4: Mean error for different averaging periods. Bar chart showing the Mean Bias between measured wind speeds and corresponding ERA5 values as a function of the temporal averaging period. Each bar represents the Mean Bias calculated for a specific averaging interval.

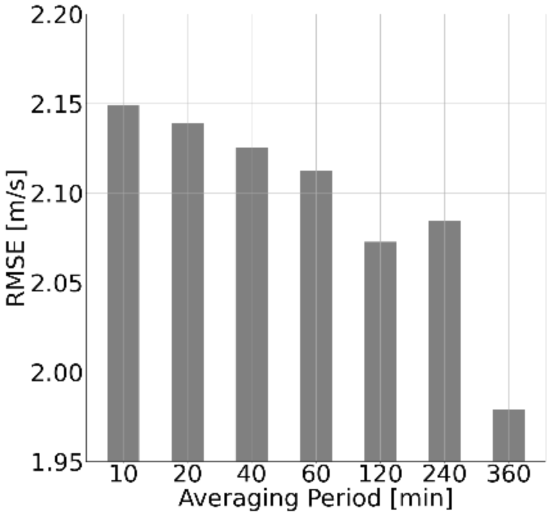

Supplementary Figure 5: RSME for different averaging periods. Bar chart showing the RMSE between measured wind speeds and corresponding ERA5 values as a function of the temporal averaging period. Each bar represents the RMSE calculated for a specific averaging interval.

1.3 Wind turbine power generation data

As the data formatting, level of detail and temporal resolution varied among the data sources, the wind turbine power generation data was harmonized in a process analogous to the wind speeds procedure mentioned above. The formatted data was also combined into one data set using the xarray python package [1].

Utilizing quality control information for the turbine data was not feasible since, in most cases, this information had been removed from the data. Therefore, the filtering process for shutdowns or curtailment due to limitations in the power grid, maintenance, or other irregularities was more demanding. These values needed to be filtered out to avoid distorting the validation results. Therefore, the following algorithm is applied to exclude out-of-normal operations.

For this, the simulation step was already performed to be able to compare measurements and simulation. First, measurements with a measured capacity factor of zero and a simultaneously simulated capacity factor of greater than 0.4 are filtered out. Second, measurements of zero capacity factor with non-changing values for more than a day are filtered out, to capture maintenance operations. Lastly, values are filtered where the measured capacity factor does not change for a minimum of 5 hours and at the same time, the difference in simulated and measured capacity factor is greater than 0.1 to filter out curtailment operations lasting longer than 5 hours.

The processing of the turbine data requires one more step. In *ETHOS.RESKit*, the hourly turbine power generation is expressed as a proportion of the turbine's nominal power where the value 1 represents the operation at full capacity and 0 no power generation. The measured values are therefore converted into this form using the formula $P(NC) = \frac{P_{turbine}}{NC_{turbine}}$ with $P_{turbine}$ as the measured power, $\mathrm{NC}_{\mathrm{turbine}}$ as the nominal capacity and $P(\mathrm{NC})$ as the converted value representing the turbine load factor. Furthermore, in case the data was on wind-park level, the reported power output was averaged to a single turbine by dividing the power output by the number of turbines in the wind park.

Finally, the processed data was saved into one netCDF file per location.

1.4 Existing wind-farms database

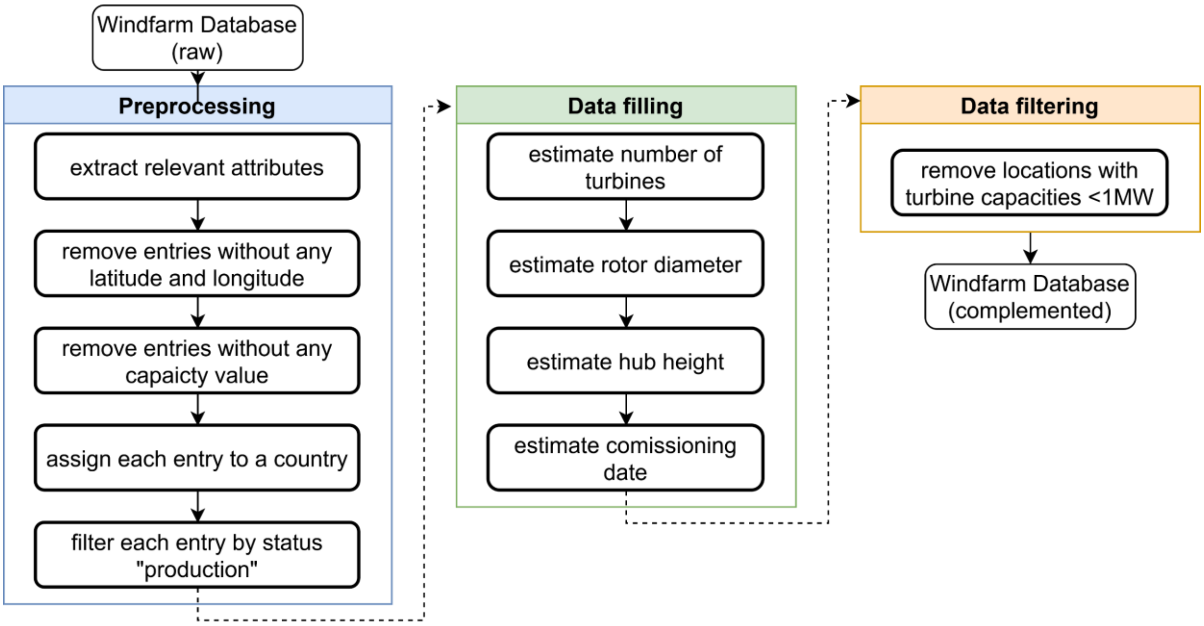

Supplementary Figure 6: Steps conducted for processing, data filling and data filtering of the wind farm database. Schematic overview of the workflow applied to the raw wind-farm database. Preprocessing: extraction of relevant attributes; removal of entries without latitude, longitude or capacity values; assignment of entries to countries; and filtering by operational status ("production"). Data filling: estimation of missing attributes, including number of turbines, rotor diameter, hub height and commissioning date. Data filtering: removal of locations with turbine capacities below 1 MW. The resulting dataset constitutes the complemented wind-farm database.

Data on existing windfarms was acquired from thewindpower.net (TWP) [2] alongside databases on turbine models and power curves.

The data contains locations of 26,900 entries on operational, planned, and decommissioned onshore and offshore wind farms worldwide. The provided data attributes include the name of the wind-park, the geolocation (lat, lon), capacity, number of turbines, hub-height, decommissioning and commissioning dates as well as the used turbine model. The turbine model database contains e.g. data on the manufacturer, rated power, rotor-diameter, and market introduction as well as minimum and maximum hub height of that model.

As not all data fields were present in the wind-farm database, the following methodology was employed for filling in missing data as well as removing unusable entries as shown in Supplementary Figure 6.

First, relevant data attributes were collected by matching the data of the turbine models to the wind-farms database. If the turbine model was known, the rotor-diameter was taken from the turbine model database.

Relevant attributes include "ID", "Latitude", "Longitude", "Turbine", "Manufacturer", "Hub height", "Total power", "Number of turbines", "Status", "Commissioning date", "Decommissioning date" and "Rotor diameter". Entries without any latitude and longitude data are removed. In a preprocessing step, the country and continent of each location is determined

using latitude and longitude to geospatially match each location to a country shapefile. GADM[3] data was used for the administrative areas of all countries, while the exclusive economic zones (marineregions.org[4]) were used to assign offshore regions to countries. The database is filtered for wind parks in operation by filtering for status “production”. Entries without a given capacity are dropped as this is the least requirement to be able to simulate the wind park.

Three parameters were estimated as follows if they were not present for a wind farm: “Number of turbines”, “Hub-height” and “Rotor diameter”:

After performing the above steps, 32402 entries are present in the database. 3686 locations had missing data on the number of turbines. The number of turbines was estimated by calculating the average number of turbines per capacity based on the entries with the available number of turbines (32402-3686 entries). Multiplying this value with the capacity of the wind park yielded the number of turbines. In a second step, missing entries for the “Rotor diameter” are estimated. 7343 locations had missing entries for the rotor diameter. First, the locations were grouped by continent. Second, a power law fit was applied to fit the capacity and rotor diameter on a continent basis. Power law fit was chosen due to the observed relationship in the rest of the dataset. We refrained from using a country-level estimation as some countries (especially with few wind farms) only have a very limited amount of entries for rotor diameters. Third, missing data on the rotor diameter was estimated by applying the power law fit using the location´s capacity. In a fourth step, missing entries for the hub-height are estimated. 11549 locations had missing entries on the hub-heights. If the turbine model was given, the mean of the minimum and maximum available hub-height of the respective turbine model was used. For the remaining entries, the hub-height is estimated using a linear fit between hub-height and rotor-diameter (as the rotor-diameter showed the highest Pearson correlation of the available parameters). A linear fit was chosen because of the observed relationship on the rest of the data for locations with a hub height smaller than half the rotor diameter, the hub height is set to half the rotor diameter (To make sure the fit stays in a technical possible limit).

If the location had an unknown commissioning date and the turbine model was given, 2 years after the market introduction was assumed as the commissioning date. Finally, if the turbine model was known, a power-curve from the power curve database was assigned (if available). Further, a last filtering step is applied in which locations with turbine capacities ≤1MW or park capacities ≤ 3MW are removed as such turbines typically have very low hub-heights which produce unrealistic simulation results in*ETHOS.RESKit*. This is primarily due to the uncertainty in the wind speeds at hub height, which results from the large scaling factors when wind speeds are scaled logarithmically from 100m height (as provided in the ERA5 data) to very low hub heights.. However, *ETHOS.RESKit* is designed for potential assessments of future energy systems. Here, wind turbines with small hub-heights will likely not play a major role. Additionally, locations with average wind speeds ≤ 3 m/s according to GWA4 are excluded as they are considered erroneous.

1.5 Country-level statistical data

As outline in the methodology, annual power generation and capacity data for historical years are obtained for onshore and offshore wind on country level from the IEA. From this, a preliminary capacity factor is calculated by dividing the reported power generation for a year by the reported capacity. Notable anomalies were found in the IEA data, especially for 2022 where unrealistic capacity additions or subtractions appeared. An example of this is Indonesia in which the capacity dropped from 0.22 GW in 2020 to 0.15 GW in 2021. Therefore, 2021 was

chosen as last year. Data was filtered for 2017 to 2021 as previous years only saw a limited global ramp-up of wind energy capacity. In case of significant capacity additions in a year, the preliminary capacity factor is not accurate as the newly added capacity is not generating electricity throughout the entire year. Therefore, capacity additions are weighted by the number of months the capacity addition contributed to the overall electricity generation. In case the reported country capacity from the wind park database showed significant deviations from the capacity reported by the IEA, commissioning dates were manually added by conducting internet research on individual wind farm projects using data from e.g.: power-technology.com[5]. This is especially necessary for countries with limited wind turbine capacities as small deviations in the data have a large impact on the reliability of the calculated capacity factor and therefore the validation results. Additionally, for every country we exclude years in which the country capacity in the wind-farms database is below 75% of the capacity reported by the IEA. Additionally, we exclude years in which the country's IEA capacity was below or equal 3 MW. The supplementary materials include a spreadsheet with all exclusions and corrections.

For European countries, we additionally calculated capacity-weighted time-resolved capacity factors based on country-aggregated hourly power generation values from the ENTSO-E transparency platform [6] as a further validation basis for our simulation results. ENTSO-E provides absolute hourly generation time-series at national or sub-national level. In case of sub-national data, the time-series were aggregated to national level through summation. As the reported capacity on the ENTSO-E transparency platform did not match the simulated country capacities in some cases, for consistency reasons the IEA capacity as ground truth for the respective year was used to calculate capacity factors. For this, the hourly power generation values were divided by the installed annual capacity reported by the IEA. It should be noted that this approach does not correctly reflect the capacity additions during a year and can therefore lead to deviations in the capacity factor. However, the data have been included as the focus of this comparison is on the correlation of the time series, which are not as prone to the aforementioned variations as the total electricity generation.

1.6 *ETHOS.RESKit* wind power simulation workflow

The methodology in *ETHOS.RESKit* for simulating wind speed and turbine power is built upon the framework described by Ryberg et al.[7] with notable enhancements. New developments include the adoption of ERA5 data instead of MERRA-2, chosen for its superior spatial resolution and wind speed height values compared to MERRA-2. Additionally, the model incorporates the latest version of the Global Wind Atlas (GWA4) with an enhanced spatial resolution of 250m², a significant improvement from the 1 km² grid spacing in the original version used by Ryberg et al.[7] and Caglayan et al.[8]. We modify the long-term average used to normalize ERA5 with GWA4 values to be in line with the GWA4 observation period. Furthermore, the applicability of the simulation workflow is extended to global scale including offshore locations.

Part of the simulation procedure was already published in Ryberg et al.[7]. However, for comprehensiveness reasons, we present the whole workflow in Supplementary Figure 8. First, all relevant turbine parameters and workflow parameters need to be specified. Turbine parameters include location, time period, hub height, rotor diameter, and capacity. Optionally, the user can provide a turbine model. If that is the case, the turbine model's power curve is used instead of a synthetic power curve [7]. It should be noted that multiple locations (up to

several thousand) can be simulated at once. Optional workflow parameters include setting an availability factor, a wake reduction curve, a country correction factor, and a wind speed calibration factor.

The simulation procedure can be summarized as follows:

1. Resampling: ERA5 wind speeds at a height of 100 m are resampled to match the grid spacing of GWA4 using linear interpolation.
2. Long-term average (LRA): A ten-year average (2008-2017, matching the exact years that were also used for the generation of GWA4 [9]) is calculated based on the hourly ERA5 data at the desired location.
3. Correction factor: The LRA is divided by the value from GWA4, resulting in a correction factor. This factor is then applied to the resampled ERA5 time series data to improve the representation of long-term microscale effects.
4. Vertical extrapolation of wind speed: The corrected wind speeds are extrapolated to the hub height (or anemometer height) using the observed shear from different height levels (10, 50, 100, 150 and 200m) of the GWA4. For this, a scaling factor is derived by calculating the wind speed at hub height from the GWA4 by interpolation divided by the wind speed from GWA4 at 100m. Simulated wind speeds are then extrapolated by multiplication with this scaling factor. Planetary boundary layer effects are also considered by only applying wind speed scaling when the target height is lower than or equal to the top of the boundary layer. If applicable, a wind speed correction is employed using wind-speed dependent regressors as described in the section "Calibration and cross-validation of estimated wind speeds from reanalysis weather data" of the main document (see also Section 1.7.1 below for background information).
5. If applicable, wind speed losses due to wake effects are considered using the wind efficiency curves from windpowerlib [10].
6. If available, the manufacturer's power curve is used, otherwise a synthetic power curve, as described in Ryberg et al. [7], is applied.
7. To calculate the power output at the turbine location, the following steps are carried out:
   a) Air density correction: The simulated wind speed is adjusted for air density based on the IEC 61499-12-1:2017 standard [11]. The air density correction is resolved hourly and based on actual wind speed, pressure at ground level, temperature, with the turbine height and sea level reference and the following constants:
      1) g0 = 9.80665 Gravitational acceleration [m/s2],
      2) Ma = 0.0289644 Molar mass of dry air [kg/mol],
      3) R = 8.3144598 Universal gas constant, [N·m/(mol·K)],
      4) rhoSTD = 1.225 Standard air density [kg/m3].
   b) Power curve convolution: The adjusted wind speed is convolved with the power curve using a scaling factor of 0.01 and a base factor of 0.
   c) Power output simulation: The power curve is applied to the simulated wind speeds to simulate the power output or capacity factors.
   d) If applicable, a power-output correction factor is employed (e.g. country correction factor or losses) that further corrects wind speeds to meet target capacity factor.

By default, we employ the wake reduction curve “knorr_mean” from the windpowerlib python package as it showed the best alignment with our results [10], as shown below in Supplementary Figure 7 based on a comparison with the real wind turbine outputs introduced in the Methods subsection “Wind turbine electricity power generation data” in the main document.

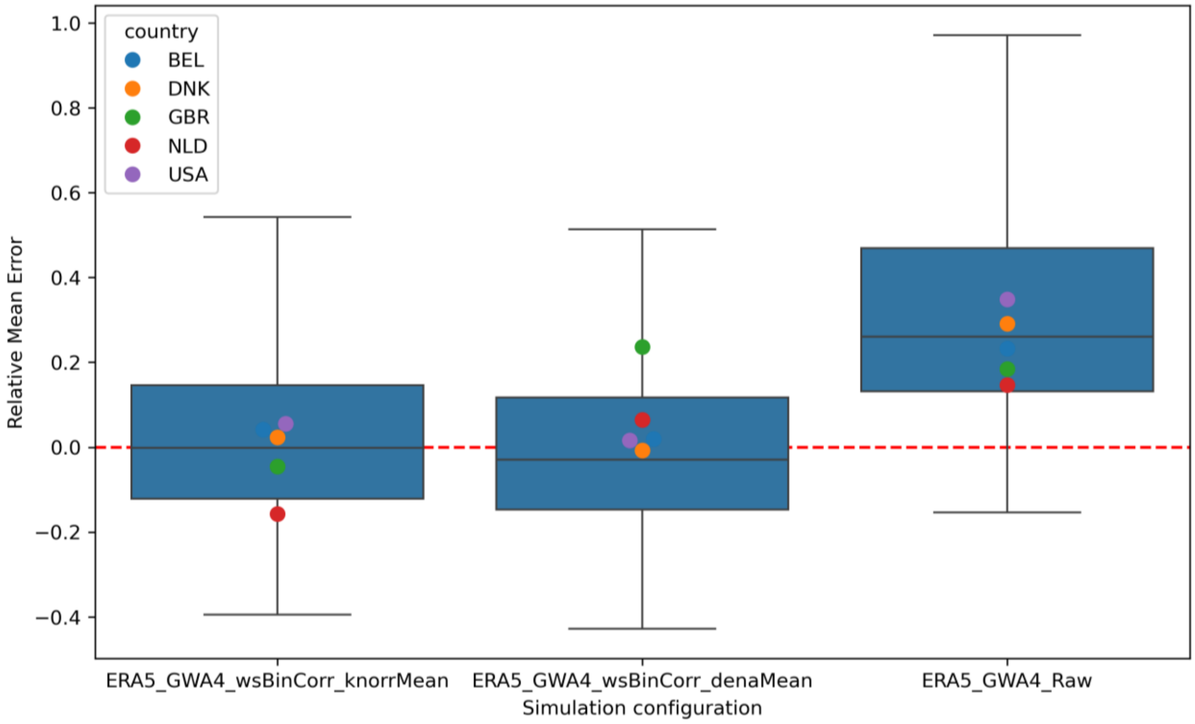

Supplementary Figure 7: Mean error for hourly wind farm outputs under consideration of different wake reduction curves compared to the uncorrected raw data. Box plots compare the distribution of Relative Mean Error (RME) for annual wind speed measurements using an uncorrected raw workflow based on ERA5 (European Centre for Medium-Range Weather Forecasts Reanalysis v5) and GWA4 (Global Wind Atlas 4) (ERA5_GWA4_Raw) and two modified simulation configurations (ERA5_GWA4_wsBinCorr_knorrMean and ERA5_GWA4_wsBinCorr_knorrMean) employing a wind speed correction and wind speed reductions from wake effects. In these plots, the center line represents the median, the box limits show the upper and lower quartiles, and the whiskers extend to 1.5 times the interquartile range. Individual data points indicate the RME for four countries: Belgium (BEL), Great Britain (GBR), the Netherlands (NLD), and the USA. The dashed red line indicates zero error. Both simulation configurations are shown to reduce the median error and overall spread of the simulations compared to the raw data. Source data are provided as a Source Data file.

Following Lee et al. and Fraunhofer ISI we employ an availability factor of 0.98 to approximate downtimes for e.g. maintenance of a turbine within a year [12,13]. No other losses such as environmental losses (icing etc.) are considered by default. Degradation is not included at this stage but is implicitly considered in the last workflow step when the annual average capacity factor is corrected to realistic outputs of an average fleet, with average age.

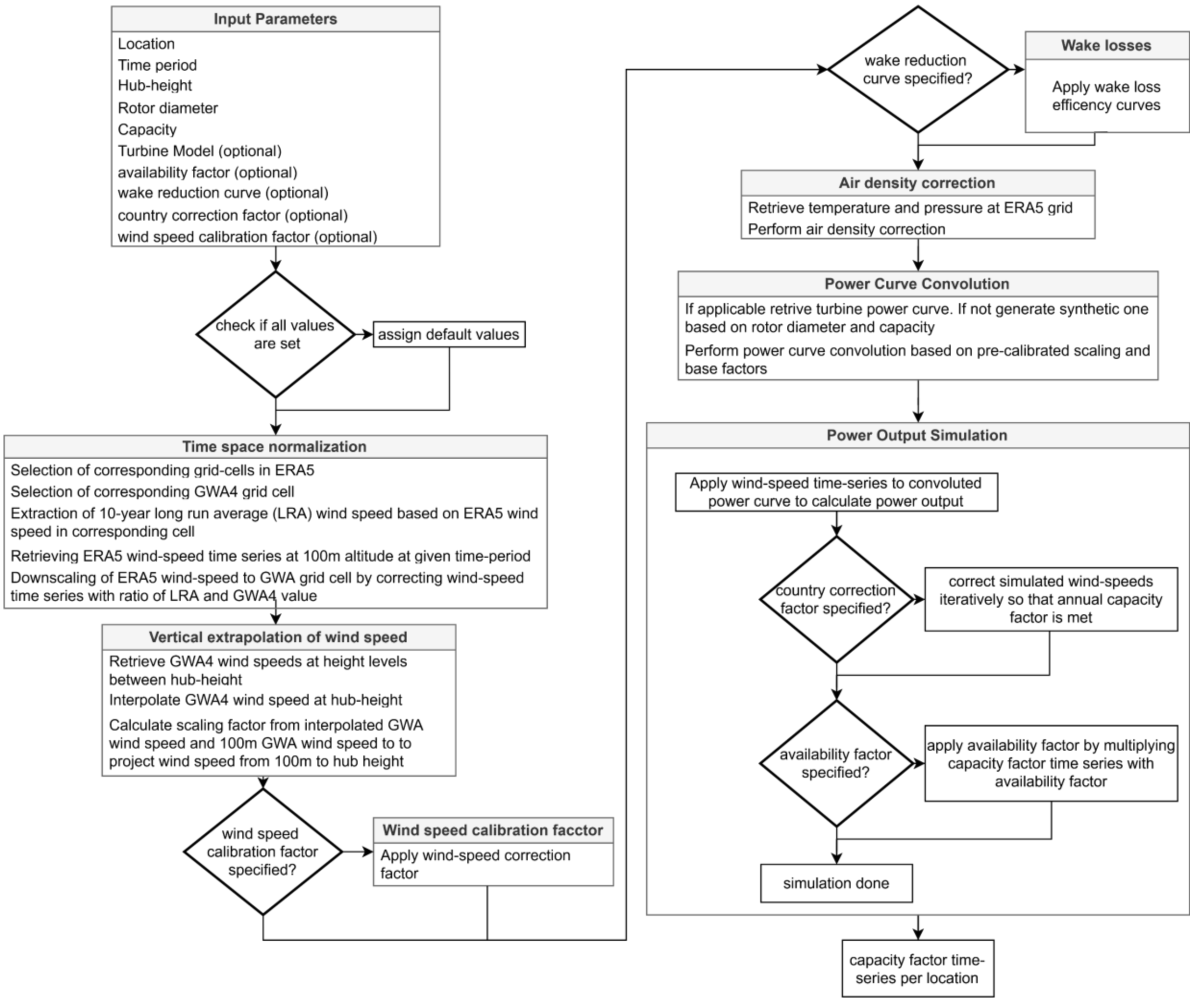

Supplementary Figure 8: *ETHOS.RESKit* power simulation workflow. Schematic overview of the steps used to simulate wind-turbine power output in *ETHOS.RESKit*. Input parameters include location, time period, turbine characteristics, and optional correction factors. After checking and assigning default values, the workflow performs time–space normalisation using ERA5 and GWA4 data, including grid-cell selection, long-run average scaling, and retrieval of height-specific wind speeds. Wind speeds are then projected to hub height and, if specified, corrected using wind-speed calibration factors. Air-density correction, wake-loss adjustment, and turbine-specific power-curve convolution are applied before calculating power output. Optional country-level correction factors and availability factors are incorporated iteratively to ensure consistency with observed annual capacity factors. The final output is a simulated capacity-factor time series for each location.

1.7 Calibration and cross-validation of reanalysis wind-speed

1.7.1 Use of alternative cross-validation regressors

As outlined in the methodology, a k-fold cross-validation with wind speed dependent regressors is used to obtain wind-speed correction factors.

In addition to this multiple regressors were tested for wind-speed correction, such as a linear regressor, a multiple linear regressor, a multiple polynomial regressor and a Multi-Layer-Perceptron (MLP) regressor.

The linear regressor was defined by a scaling factor *a* and offset factor *b* and underwent the same procedure as described in the methodology. While the linear regressor showed good results on average it performed worse than the wind speed based proportional regressor. The resulting linear resulted in scaling and offset factors of *a = 0.751* and *b = 0.906 m/s*. As a result, high wind speeds were corrected strongly, leading to underestimation in high wind speed regions such as the North Sea. Therefore, the linear regressor was discarded.

The further regressors were trained on additional input data such as land cover, surface roughness, height above ground, solar elevation, month of the year as well as latitude and longitude with the goal of addressing additional spatial and temporal mean errors present in the ERA5 data.

A multiple linear regressor was tested by the same procedure outlined in the methodology. In addition to the modelled wind speed, this regressor was tested with the ESA CCI land cover code, the resulting surface roughness, height above ground, solar elevation at the given time and position, solar time, month of the year as well as latitude and longitude as additional input parameters for the regressor in all possible combinations. The best combination was found to be the ESA CCI land cover code and latitude as additional parameters for the linear correction of the modelled wind speed. The solar time is generated by the solar elevation so that it is set to hour zero with sunrise.

The second method uses a multiple polynomial regressor. Unlike the linear regressor, the polynomial regressor has the advantage of being able to correct for non-linear mean errors. While this regressor offers advanced possibilities, it also increases the risk of overfitting to the specific dataset. This regressor was fitted and validated in the same cross-validation with the addition of a multi-parameter grid search to determine the best combination of input parameters, as well as the degree of the polynomial function. The best results were obtained with the ESA-CCI landcover code, surface roughness, solar time and elevation and month of the year as parameters, and a fifth degree regressor.

The third method is the usage of a Multi-Layer-Perceptron (MLP) regressor. It was trained and validated in the same cross-validation procedure. In a grid search a wide range of hyperparameters, as well as the possible variations of input parameters was tested. As the largest deviations occur at the spatial level, latitude and longitude were initially included in the input parameters to correct for spatial mean errors.

However, it was observed that the utilization of the additional regressors did lead to notable anomalies in the corrected wind speeds showing signs of overfitting. E.g. in the case of the MLP regressor, the regressor exhibited a tendency to overly adjust the wind speed based on the specific geographic coordinates, which resulted in reduced generalization capabilities. Another issue was excessive overcorrection of particularly high wind speeds, reducing them by up to 8m/s. Hence, it was decided that the alternative regressors would not be further considered in the subsequent analysis and evaluation.

1.8 National correction factors

As mentioned in the Paper, the national correction factors are included as Supplementary Data 2. Moreover, the correction factor raster file is available as Supplemenatry Data 4 and in the ETHOS.*RESKit* GitHub repository [14].

1.9 ENTSO-E wind power generation time-series comparison

Supplementary Figure 9 compares country-level hourly power generation data from ENTSO-E and our simulations for 2017-2021 using the DCCA coefficient to assess correlation. The methodology is described in section 1.5. Most countries show DCCA coefficients above 0.9, indicating a strong match with *ETHOS.RESKit* results, though some coefficients drop as low as 0.69. This discrepancy primarily stems from three causes: (1) differences in wind fleet capacity between our simulations and ENTSO-E, as ENTSO-E lacks capacity time series data, leading us to rely on IEA capacity values; (2) assumptions about wind turbine characteristics, such as model type, height, and commissioning dates, which are challenging to align precisely; and (3) external factors like grid congestion, curtailment, and accounting for imports/exports introduce further differences not captured in our model. These assumptions, coupled with imprecise data records, challenge direct comparisons. Despite these limitations, most countries display a strong alignment between simulated and ENTSO-E data, demonstrating the capability of *ETHOS.RESKit* to accurately simulate country-level wind power generation.

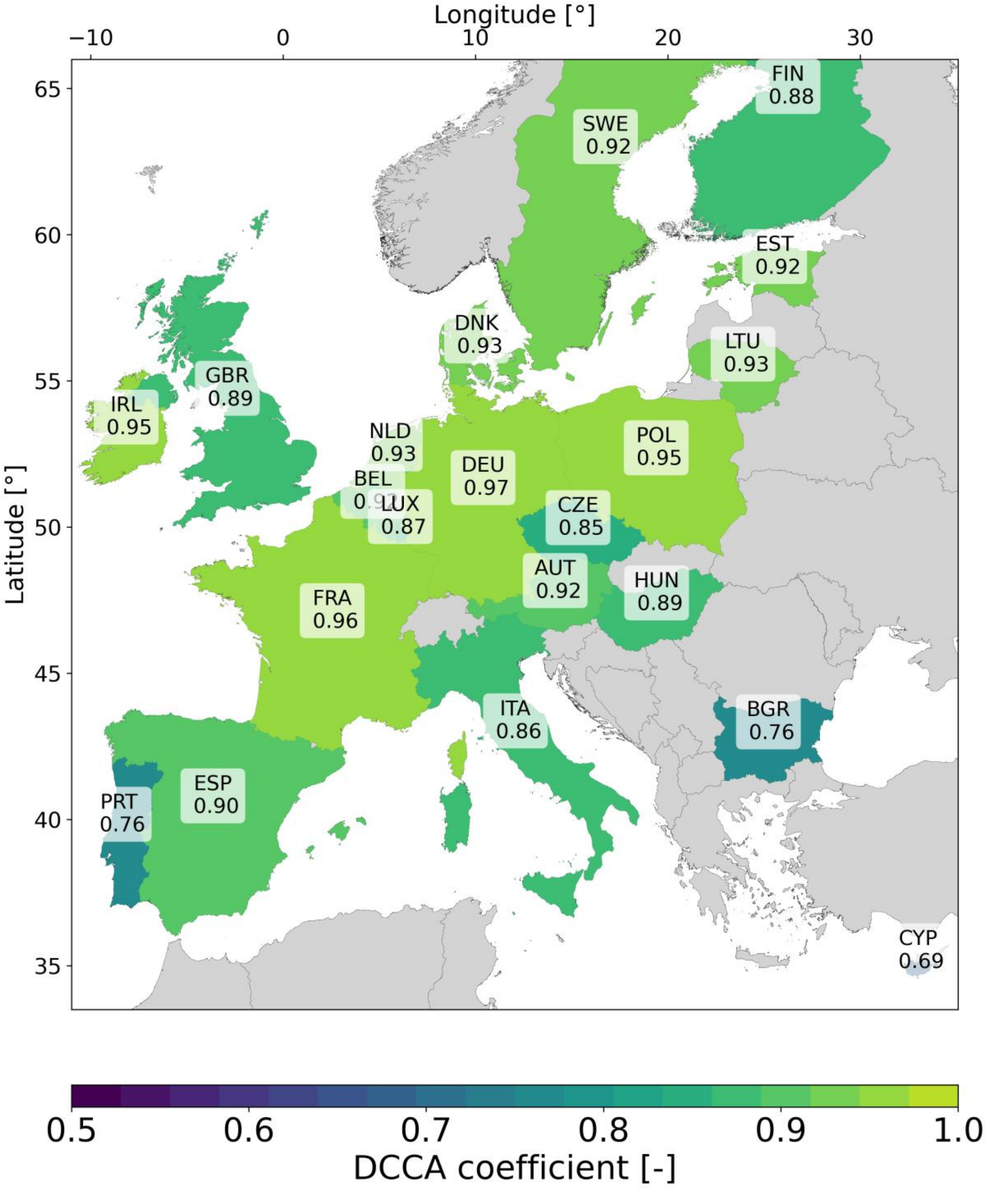

Supplementary Figure 9: Detrended Cross-Correlation Analysis (DCCA) Coefficient between *ETHOS.RESKit* simulation and ENTSO-E publicly available data between 2017-2021. Map showing the DCCA coefficient between *ETHOS.RESKit* simulations and ENTSO-E publicly available national wind power generation data for the years 2017–2021. Colors indicate the DCCA coefficient, with higher values representing stronger correlation. Country labels report the coefficient for each country. Grey areas denote countries without data. Country shapes from GADM [3]. Source data are provided as a Source Data file.

1.10 Model performance limitations

In this section, we will delineate the identified limitations that were uncovered throughout our study. The intention behind this is to assist in narrowing the performance gaps that have been identified and to provide further insight into the interpretation of the results for those who are more experienced in this field. Firstly, while *ETHOS.RESKit* generally reduces the deviation in comparison to localized time-resolved wind power generation data, it is notable that there is a tendency for the generation to be underestimated in comparison to aggregated wind power generation at a country level. This discrepancy arises from the fact that aggregating a year's worth of power generation data into a single annual value for each country impedes the precise comparison of the power generation simulations. As illustrated in the results, this is particularly crucial when portraying high-capacity factors, as they account for the majority of power generation. Despite the implementation of measures to enhance the accuracy of aggregated results, it is possible that they may not fully replicate out-of-normal operations in all cases. Therefore, it is recommended that comparisons of model results be made against time-resolved measurements whenever feasible. Secondly, although wind power developers typically favor locations with favorable conditions and high wind potential, complex terrain types such as mountains, forests, and urban areas frequently result in significant discrepancies in power generation compared to these optimal locations, exhibiting both positive and negative deviations. This discrepancy may be attributed to the limitations of the Global Wind Atlas v4.0 (GWA4) or ERA5 data in such terrain types, as evidenced by the literature review. Additionally, diurnal and seasonal mean capacity factor deviations were observed. The impact of such wind speed mean errors is direct, affecting the performance of the model. The importance of accurately determining wind speed is evident from the wind energy power formula and is confirmed by this study. Thirdly, the limiting factor for further improvement is the availability of wind speed measurements at the hub height of the wind turbines. This is particularly relevant in the context of locational mean error correction. Should further data become available, additional regional or alternative wind speed calibration procedures may be feasible. Fourthly, it was observed that the simulation of contemporary wind turbine fleets generally produces results that are closer to the expected aggregated values for power generation. In contrast, simulations of wind turbines with a capacity below 1 MW tend to exhibit suboptimal performance, primarily due to observed discrepancies in synthetic power curve representation - since the synthetic power curve is based on 106 modern wind turbines with a capacity of at least 1 MW - and the inherent difficulties in reducing wind speeds to a scale commensurate with the terrain.

1.11 Further recommendations for model users

The *ETHOS.RESKit* model is best suited for simulating the regional wind potential from multiple turbines distributed across the region. For more detailed simulations of individual turbines, it is recommended that other measures be employed in conjunction with the aforementioned simulations. These additional measures may include the use of weather data derived from atmospheric models at the mesoscale or microscale, local wind corrections, local environmental and operational restrictions, and so forth. In the event that the simulation results are to be compared against further measured data, it is recommended that the filtering procedures described in the Methods be undertaken in order to remove data from normal operational times. It is crucial to select the optimal wind turbine design, as this choice can significantly influence both power generation and the levelized cost of energy (LCOE). Furthermore, the wake effect or technical availability factors can be modified or omitted according to the specific simulation scenario, such as the number of turbines, the theoretical

versus real performance, and so forth. In the context of country assessments, it is recommended that the results be corrected using the corresponding regional correction factors provided in supplementary material 1.8. These factors offer a comprehensive correction to account for non-physical factors influencing a region's wind power generation, based on the latest publicly available data. Depending on the specific needs of the modeler and the availability of measured data, it may be beneficial to follow a regional calibration procedure for wind speeds, based on the cross-calibration procedure presented in our study.

1.12 Additional discussion on the wind speed calibration procedure.

This subsection delves into additional discussion points regarding the wind speed calibration of the model, which the authors find relevant.

Firstly, it is clear throughout the results section that an accurate depiction of wind speeds is crucial for the model's performance. However, it was not possible to gather wind speeds data at wind turbine sites. As explained in section 2.1, the majority of wind speed measurements used for calibrating our workflow come from weather masts rather than actual wind turbine measurements. These masts are frequently positioned in areas characterized by intricate atmospheric conditions, such as mountainous regions, coastal areas, or urban environments, primarily to collect wind data for purposes other than assessing regions with consistent, foreseeable, or exploitable wind energy resources. Additionally, meteorological towers are not necessarily situated in areas with high wind speeds, whereas wind turbines are typically placed in regions with the highest possible wind speeds. As a result, the regressors trained on this data may not ideally match the conditions and wind speed velocities of actual wind turbine. One potential approach to better tailor the data to turbines is to exclude wind speeds from masts located in areas with low wind speeds or at measuring heights uncommon for wind turbines. However, this filtering would further reduce the amount of data used for calibration and may diminish the generalization properties of the regressor. In essence, the calibration procedure involves a tradeoff, improving performance for most locations at the expense of misrepresenting some locations. For the reasons mentioned, the potential of calibrating based on turbine data should also be investigated further. Nevertheless, the even more limited availability and quality of turbine power generation data presents the main obstacle for an approach like this. It is because of this lack of data that attention was shifted to the development of a calibration procedure focused on wind speed correction due to its potential to incorporate a larger number of measurements in diverse locations. The availability of increasingly relevant wind speeds, power generation data and turbine characteristics will enhance the calibration procedure.

Secondly, this study assessed various calibration methods, including Spline, Polynomial, and Multi-Layer Perceptron (MLP), but none yielded superior results. This lack of success may be attributed to overfitting to the wind speed data and an insufficient amount of measurement data. All tested regressors exhibited excessive reduction of wind speeds as correction at high wind speeds of 10 m/s and above, resulting in an increase in Mean Absolute Error (MAE) compared to uncalibrated values at these wind speeds. While this behavior is undesirable, it is likely due to the infrequency of these high wind speeds in the data. To address these issues, it is suggested that the correction by the regressors should be gradually reduced from this wind speed onwards. While a more sophisticated correction technique beyond linear calibration might offer greater effectiveness in mean error and error correction overall, linear calibration

significantly reduced general overestimations in wind speed ranges relevant for wind turbine applications. Moreover, its quick implementation makes it suitable for large-scale usage. However, it's important to note that the linear calibration procedure should not be seen as an algorithm to "correct" ERA5 wind speed data. The authors emphasize the necessity for further advancement in reanalysis weather data models to mitigate mean errors and enhance wind energy modeling.

While investigating potential biases in ERA5 the authors utilized the HadISD (Hadley Integraded Surface Database) dataset [15,16] (v. 3.3.0.2022f) containing wind speed measurements of global stations at 10m and compared it with ERA5 wind speeds at 10m. The DCCA coefficient was calculated for every station. When averaging the DCCA coefficients at different latitudes, a clear decrease of the DCCA coefficient towards the equator was observed as shown in Supplementary Figure 10, pointing at further potential biases within ERA5 not yet addressed by literature.

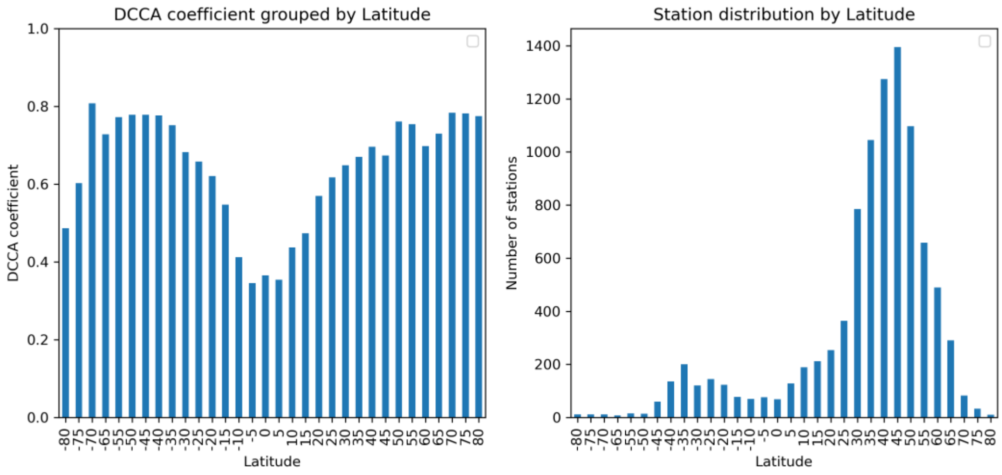

Supplementary Figure 10: DCCA coefficient and station distribution for different latitudes calculated from 10m station measurements. (Left) Mean Detrended Cross-Correlation Analysis (DCCA) coefficient between 10 m wind-speed measurements from the HadISD dataset and 10 m ERA5 wind speeds, grouped by latitude. (Right) Number of stations in the HadISD dataset available in each latitude band. Source data are provided as a Source Data file.

The comparison also revealed error trends in mean wind speeds in GWA4 10m data depending on the ruggedness of the terrain as well as on the land cover type and latitude (see Supplementary Figure 12 and Supplementary Figure 11): Supplementary Figure 11 reveals a similar pattern as Supplementary Figure 10. Especially near the equator the mean measured wind speed and the mean wind speed reported by the GWA4 differ by more than 67%. Generally, the mean wind speeds as reported by the GWA4 tend to be lower than the measured values. Differences in accuracy of the GWA4 depending on the land type can also be observed. While the GWA4 underestimates wind speeds in e.g. forest and urban terrain, wind speeds in areas with snow are greatly overestimated.

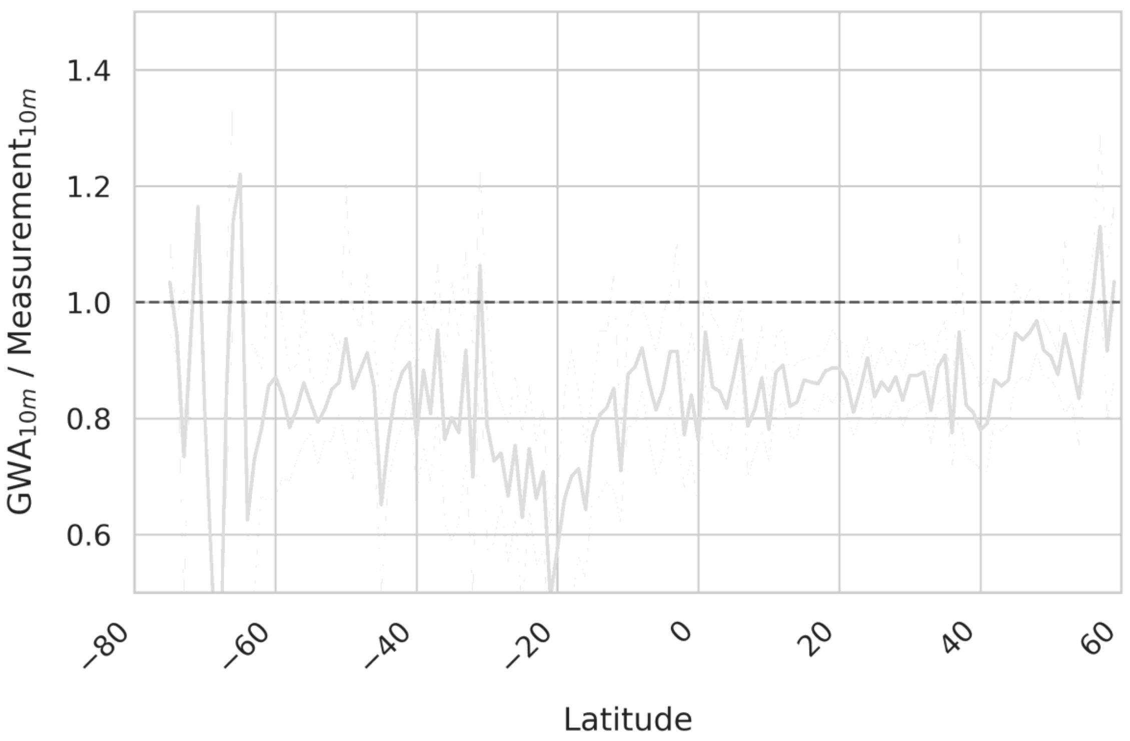

Supplementary Figure 11: Deviation between the average wind speed from 10m station measurements from HadISD dataset and corresponding GWA4 values for latitudes. Plot showing the ratio of GWA4 10 m wind speeds to 10 m wind-speed measurements from the HadISD dataset as a function of latitude. The solid line gives the mean ratio within each latitude band, and the grey shaded area shows the spread of values (interquartile range) across all stations in that band. Ratios below 1 indicate underestimation by GWA4, and ratios above 1 reflect overestimation. Source data are provided as a Source Data file.

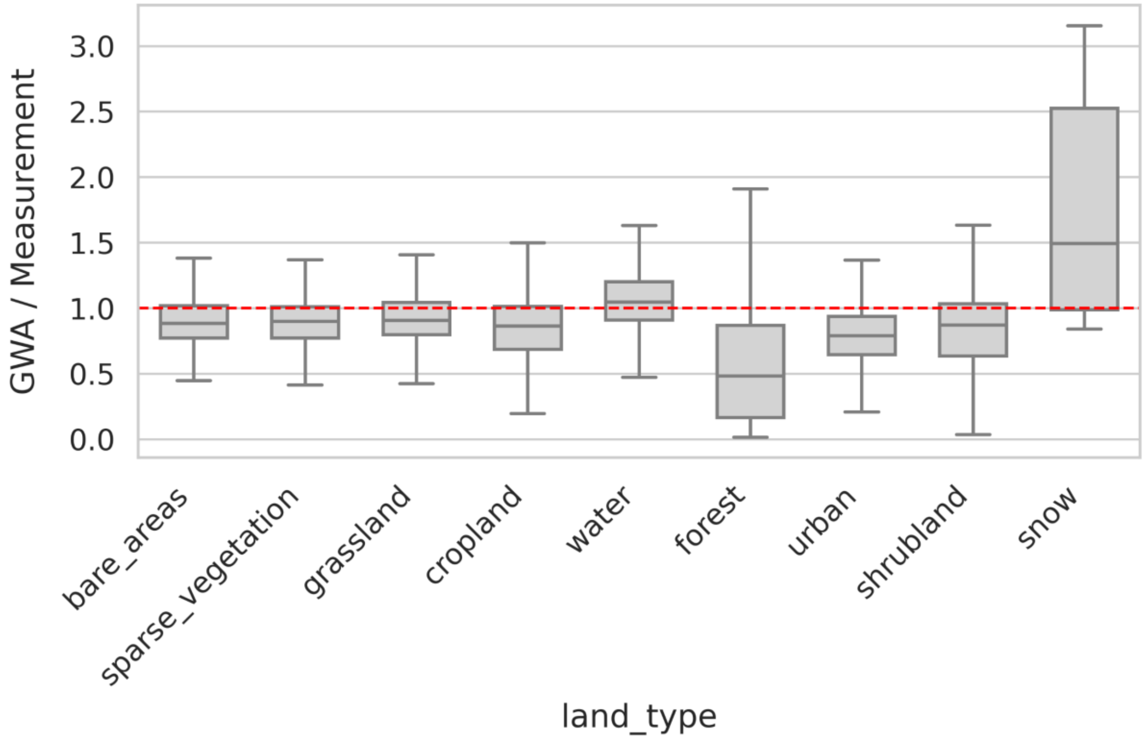

Supplementary Figure 12: Deviation between the average wind speed from global 10m station measurements from HadISD dataset and corresponding GWA4 values for different land types. Boxplots showing the ratio of GWA4 10 m wind speeds to 10 m wind-speed measurements from the HadISD dataset for each land-cover type. Grey boxes indicate the interquartile range, whiskers show the 5–95% range, and the red dashed line marks a ratio of 1.0. Source data are provided as a Source Data file.

Finally, the validation and calibration of model outcomes, though essential, are often secondary due to the significant time and human capital required. While the applicability of the models beyond Europe is acknowledged, their validity in other regions is not addressed. The authors recognize that overcoming this challenge can be difficult for many organizations and researchers with limited resources and time-resolved data.

1.13 Comparison of our model results against similar models

Disclaimer: The following results were obtained using a previous version of the ETHOS.RESKit workflow using GWA3.

We evaluated our wind power workflow of *ETHOS.RESKit* against *RenewablesNinja* and *EMHIRES* [17]. It's noteworthy that while globally applicable *RenewablesNinja* operate on MERRA2, while *ETHOS.RESKit* relies on ERA5. Furthermore, we compare our results against the *EMHIRES* [17] dataset, which is limited to Europe and provides wind turbine electricity power generation time series at NUTS2 (Nomenclature of Territorial Units for Statistics) level based on MERRA2. The *atlite* model [18] and pyGRETA were not considered in the comparison due constrains by the authors to replicate its intended performance. For the atlite

model the main challenges were related to ERA5 downloading processing time and the appropriate turbine determination. *The open-source model pyGRETA* is not tailored towards simulating individual wind parks with different characteristics including turbine specific power curves. While the authors modified the source code such that single wind farms with different specifications could be simulated, it was concluded that the necessary modifications left too much room for potential errors.

Simulations of individual wind turbines and wind farms were executed across the selected tools, and subsequent results were juxtaposed against measurement data. Various metrics, such as Mean, DCCA coefficient, and Mean Error (ME), calculated per location, were employed to gauge the quality of the simulations. It is imperative to acknowledge that not all locations and measured times could be simulated with each tool by the author, only 22 locations were common amongst all the three models. A wider range of locations would have been possible if deviations from the exact turbine model or even synthetic power curves had been accepted, however, the exact match has been selected here to exclude potential biases against models with lesser turbine model coverage. Supplementary Figure 13 shows that *RenewablesNinja*, *EMHIRES*, and *ETHOS.RESKit* produce mean capacity factors within 1% of the expected measurements. Supplementary Figure 13 provides supplementary insight by presenting a time series evaluation. A comparative analysis of *RenewableNinjas* and *ETHOS.RESKit* demonstrate comparable value ranges, with the latter exhibiting slight improvements in both the Pearson correlation (0.02), DCCA score (0.017), Perkins skill score (0.9) and root mean square error (0.015), indicating enhanced time series correlation.

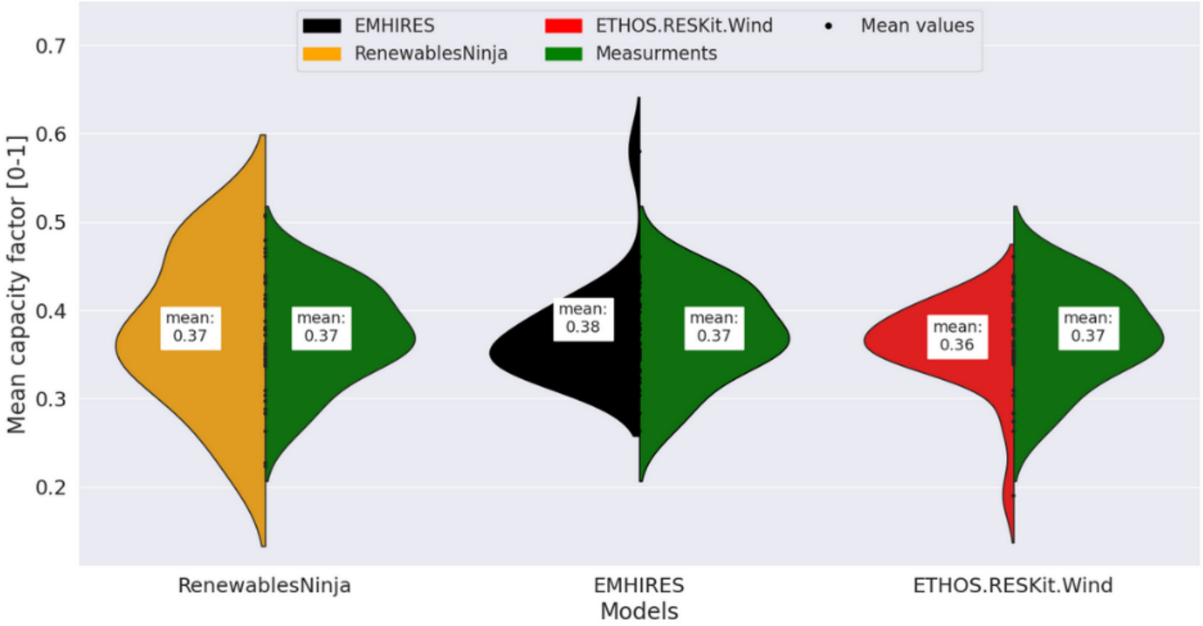

Supplementary Figure 13: Mean capacity factor comparison between 22 locations simulated using similar models. Violin plots comparing mean wind-power capacity factors from three modelling approaches—RenewablesNinja (orange), EMHIRES (black) and *ETHOS.RESKit* (red)—against measured capacity factors (green) for 22 locations. Each violin shows the distribution of mean capacity factors simulated or observed at the same sites. White labels report the mean value for each dataset, and black dots indicate the mean simulated capacity factor for each individual location.

Supplementary Table 2: Statistical indicators comparing several hourly time series wind energy simulation model results and measurements in 22 wind parks in Europe

Indicator [unitless]	*EMHIRES*	*ETHOS.RESKit*	*RenewableNinjas*
**Root-mean square error**	0.227	0.149	0.165
**Pearson correlation**	0.768	0.897	0.878
**Detrended cross-correlation analysis (DCCA) coefficient**	0.654	0.847	0.819
**Perkins skill score**	0.8663	0.8754	0.7810
**Mean error**	-0.0020	-0.0123	0.0031
**number of locations**	22		
**total amount observations [years]**	98		

In summary, the aforementioned findings suggest that the previously observed enhancements also result in superior statistical indicators in comparison to similar models. We declare that we have implemented each model to the best of our abilities to replicate its intended performance. However, this comparison has limitations and should not be regarded as a definitive evaluation of the models' performance.

Locational and turbine model information about the particular wind farms used for the above comparison are part of the proprietary dataset that can be purchased from thewindpower.net, so the exact locations and designs cannot be shared here. To enable reproducibility of the results for users with access to thewindpower.net data, the following Table contains the indicative locations of the wind farms as well as the capacity.

Supplementary Table 3: Indicative locations and capacities of the wind farms used for comparison

Wind farm No.	Latitude [°]	Longitude [°]	Turbine capacity [kW]
1	64.9015	10.8888	2300
2	63.5012	8.7682	4200
3	57.2590	9.6637	3300
4	57.2479	9.6826	3300
5	55.1458	11.9620	3300
6	55.1480	11.9586	3300
7	59.3157	4.9048	600
8	54.8082	11.5000	3300
9	58.1557	6.6818	2300
10	56.4970	9.1926	3000
11	56.5038	9.2013	3000
12	64.2248	10.3746	2300
13	54.8384	11.3002	3000
14	56.5004	9.1969	3000
15	58.7304	5.9481	3450
16	56.7656	8.6697	3000
17	63.8198	9.6300	2300
18	64.2155	10.4167	3300
19	54.8137	11.4983	3300
20	54.8318	11.3026	3000
21	55.1435	11.9654	3300
22	56.7567	8.6451	3000

1.14 Data sources

Supplementary Table 4. Data sources used in this study.

Data source name	Type of data	Number of locations used	period [start-end]	Original resolution	Measurement heights [min-max]	Capacity	Rotor diameter	Spatial coverage	Source
**The Tall Tower Dataset**	Meteorological Data	174	1983-2021	10min-1h	2-488	n.a.	n.a.	World	Ramon et al. [19]
**ICOS**	Meteorological Data	23	2015-today	10min-1h	5-341	n.a.	n.a.	Europe	ICOS Atmosphere Level 2 data[20]
**NEWA**	Meteorological Data	2	2016-2017	20Hz-10min	60-135	n.a.	n.a.	Europe	[21,22]
**Jülich Research Center**	Meteorological Data	1	1981-2020	10 min	100-120	n.a.	n.a.	Germany	Personal correspondence
**Norwegian government agency NVE**	Turbine Data	28	19 years	1h	46-149	600-5700	44-149	Norway	[23]
**New Zealand Electricity Authority EMI**	Turbine Data	5	10-16 years	1h	65-80	1600-3000	70-100	New Zeeland	[24]
**Fraunhofer Institute**	Turbine Data	9	2-3 years	1h	67-111	2300-6150	93-154	Germany	Personal correspondence
**Danish Energy Agency**	Turbine Data	102	6 years	1h	80-120	3000-8600	90-167	Denmark	[25]
**Pedra do Sal and Beberibe Wind Farm**	Turbine Data	20	2 years	10min	55	900	44	Brazil	[24] ([26])
**Denker and Wulf**	Turbine Data	8	2 years	1h	128-141	2300-6150	92-141	Germany	Personal correspondence
**Wind Farm Database**	Global wind farms	26900	n.a.	n.a.	n.a.	n.a.	n.a.	World	TWP[2]
**TurbineType Database**	database	n.a.	n.a.	n.a.	n.a.	n.a.	n.a.	n.a.	TWP[2]
**Power Curve Database**	database	n.a.	n.a.	n.a.	n.a.	n.a.	n.a.	n.a.	TWP[2]
**National wind power generation and capacity**	statistical data	143 Countries	2017-2021	Yearly	n.a.	n.a.	n.a.	World	IEA[27]
**National time series of wind power generation**	time series	23 Countries	2017-2021	1h	n.a.	n.a.	n.a.	EU	ENTSOE[6]

Supplementary References

[1] S. Hoyer, J. Hamman, xarray: N-D labeled Arrays and Datasets in Python, Journal of Open Research Software 5 (2017). https://doi.org/10.5334/jors.148.

[2] The wind power, World wind farms database, (2023). https://www.thewindpower.net/.

[3] GADM, Database of Global Administrative Areas, (2023). https://gadm.org.

[4] Marine Regions, (2024). https://marineregions.org/ (accessed March 25, 2024).

[5] Power Technology, Power plant profile: Jelovaca Wind Farm, Bosnia and Herzegovina, Power Technology (2022). https://www.power-technology.com/marketdata/power-plant-profile-jelovaca-wind-farm-bosnia-and-herzegovina/ (accessed March 25, 2024).

[6] ENTSO-E Transparency Platform, (2024). https://transparency.entsoe.eu/ (accessed March 8, 2024).

[7] D.S. Ryberg, D.G. Caglayan, S. Schmitt, J. Linßen, D. Stolten, M. Robinius, The future of European onshore wind energy potential: Detailed distribution and simulation of advanced turbine designs, Energy 182 (2019) 1222–1238. https://doi.org/10.1016/j.energy.2019.06.052.

[8] D.G. Caglayan, D.S. Ryberg, H. Heinrichs, J. Linßen, D. Stolten, M. Robinius, The techno-economic potential of offshore wind energy with optimized future turbine designs in Europe, Applied Energy 255 (2019) 113794. https://doi.org/10.1016/j.apenergy.2019.113794.

[9] N.N. Davis, J. Badger, A.N. Hahmann, B.O. Hansen, N.G. Mortensen, M. Kelly, X.G. Larsén, B.T. Olsen, R. Floors, G. Lizcano, P. Casso, O. Lacave, A. Bosch, I. Bauwens, O.J. Knight, A.P. van Loon, R. Fox, T. Parvanyan, S.B.K. Hansen, D. Heathfield, M. Onninen, R. Drummond, The Global Wind Atlas: A High-Resolution Dataset of Climatologies and Associated Web-Based Application, Bulletin of the American Meteorological Society 104 (2023) E1507–E1525. https://doi.org/10.1175/BAMS-D-21-0075.1.

[10] S. Haas, U. Krien, B. Schachler, S. Bot, kyri-petrou, V. Zeli, K. Shivam, S. Bosch, wind-python/windpowerlib: Silent Improvements, (2021). https://doi.org/10.5281/zenodo.4591809.

[11] International Electrotechnical Commission, Wind energy generation systems. Part 12-1 Power performance measurements of electricity producing wind turbines = Systèmes de génération d’énergie éolienne. Partie 12-1, Mesures de performance de puissance des éoliennes de production d’électricité (IEC 61400-12-1), IEC, Geneva, 2017.

[12] J.C.Y. Lee, M.J. Fields, An overview of wind-energy-production prediction bias, losses, and uncertainties, Wind Energ. Sci. 6 (2021) 311–365. https://doi.org/10.5194/wes-6-311-2021.

[13] Fraunhofer ISI, consentec, ifeu, TU Berlin, Langfristszenarien für die Transformation des Energiesystems in Deutschland, (2022). https://langfristszenarien.de/enertile-explorer-wAssets/docs/LFS3_T45_Webinar_Angebot_Nov_2022_final_webinarversion.pdf (accessed December 15, 2023).

[14] FZJ-IEK3, RESKit - Renewable Energy Simulation toolkit for Python, (2023). https://github.com/FZJ-IEK3-VSA/RESKit (accessed June 9, 2023).

[15] R.J.H. Dunn, K.M. Willett, D.E. Parker, L. Mitchell, Expanding HadISD: quality-controlled, sub-daily station data from 1931, Geoscientific Instrumentation, Methods and Data Systems 5 (2016) 473–491. https://doi.org/10.5194/gi-5-473-2016.

[16] R.J.H. Dunn, K.M. Willett, P.W. Thorne, E.V. Woolley, I. Durre, A. Dai, D.E. Parker, R.S. Vose, HadISD: a quality-controlled global synoptic report database for selected variables at long-term stations from 1973–2011, Climate of the Past 8 (2012) 1649–1679. https://doi.org/10.5194/cp-8-1649-2012.

[17] European Commission. Joint Research Centre., EMHIRES dataset. Part I, Wind power generation., Publications Office, LU, 2016. https://data.europa.eu/doi/10.2790/831549 (accessed August 31, 2023).

[18] F. Hofmann, J. Hampp, F. Neumann, T. Brown, J. Hörsch, atlite: A Lightweight Python Package for Calculating Renewable Power Potentials and Time Series, JOSS 6 (2021) 3294. https://doi.org/10.21105/joss.03294.

[19] J. Ramon, L. Lledó, N. Pérez-Zanón, A. Soret, F.J. Doblas-Reyes, The Tall Tower Dataset: a unique initiative to boost wind energy research, Earth System Science Data 12 (2020) 429–439. https://doi.org/10.5194/essd-12-429-2020.

[20] D. Kubistin, C. Plaß-Dülmer, S. Arnold, T. Kneuer, M. Lindauer, J. Müller-Williams, ICOS RI, ICOS Atmosphere Level 2 data, Steinkimmen, release 2023-1, (2023). https://doi.org/10.18160/BJ1Z-BE0T.

[21] M. Dörenkämper, B.T. Olsen, B. Witha, A.N. Hahmann, N.N. Davis, J. Barcons, Y. Ezber, E. García-Bustamante, J.F. González-Rouco, J. Navarro, M. Sastre-Marugán, T. Sīle, W. Trei, M. Žagar, J. Badger, J. Gottschall, J. Sanz Rodrigo, J. Mann, The Making of the New European Wind Atlas – Part 2: Production and evaluation, Geoscientific Model Development 13 (2020) 5079–5102. https://doi.org/10.5194/gmd-13-5079-2020.

[22] A.N. Hahmann, T. Sīle, B. Witha, N.N. Davis, M. Dörenkämper, Y. Ezber, E. García-Bustamante, J.F. González-Rouco, J. Navarro, B.T. Olsen, S. Söderberg, The making of the New European Wind Atlas – Part 1: Model sensitivity, Geoscientific Model Development 13 (2020) 5053–5078. https://doi.org/10.5194/gmd-13-5053-2020.

[23] Produksjonsrapporter - NVE, (2024). https://www.nve.no/energi/energisystem/vindkraft/produksjonsrapporter/ (accessed March 25, 2024).

[24] K. Gruber, P. Regner, S. Wehrle, M. Zeyringer, J. Schmidt, Towards global validation of wind power simulations: A multi-country assessment of wind power simulation from MERRA-2 and ERA-5 reanalyses bias-corrected with the global wind atlas, Energy 238 (2022) 121520. https://doi.org/10.1016/j.energy.2021.121520.

[25] Historic data of wind turbine installations in the whole of Denmark from the Danish Energy Agency (Energistyrelsen), (2019). https://doi.org/10.11583/DTU.7599698.v1.

[26] J. Passos, Y. Sakagami, P. Santos, R. Haas, F. Taves, Costal operating wind farms: two datasets with concurrent SCADA, LiDAR and turbulent fluxes, (2017). https://doi.org/10.5281/zenodo.1475197.

[27] Renewable Energy Progress Tracker – Data Tools, IEA (2024). https://www.iea.org/data-and-statistics/data-tools/renewables-data-explorer (accessed March 25, 2024).
