# Supplementary material for: Towards high resolution, validated and open global wind power assessments
Source: Nat Commun. 2026 Jan 14;17:539. doi: 10.1038/s41467-026-68337-z (PMC12804690; doi:10.1038/s41467-026-68337-z)
Supplement: Supplementary file 2 — Description of Additional Supplementary Files [file 41467_2026_68337_MOESM2_ESM.pdf]

Description of Additional Supplementary Files

Supplementary Data 1. Wind speed correction factors. This file contains the wind speed dependent wind speed correction factors derived from calibration using measured wind speeds and the reanalysis wind speeds from ERA5 (including GWA4 - downscaling). The factors correct for systematic mean errors and under - or overestimations.

Supplementary Data 2. Country capacity factor correction factors. This dataset provides the derived national capacity factor correction factors that adjust capacity factors simulated with ETHOS.RESKit to match observed national statistics.

Supplementary Data 3. Modifications to thewindpower.net wind -farm database. This file lists the curated corrections applied to the proprietary wind farm database from thewindpower.net. It includes corrections for location, hub height and commissioning date. Additionally, it lists the exclusions of countries and or single years for several countries for the national IEA statistics. Reasons for exclusion are documented.

Supplementary Data 4. Global raster of capacity factor correction factors. The file provides a global raster with capacity factor correction factors for ETHOS.RESKit to align with national statistics, enabling assessments in regions without wind production and enhancing global. This additional option in the presented workflow allows the simulation of wind turbines in any country of the world with realistic average capacity factors.
