# Supplementary figures and images for: Towards high resolution, validated and open global wind power assessments
Source: Nat Commun. 2026 Jan 14;17:539. doi: 10.1038/s41467-026-68337-z (PMC12804690; doi:10.1038/s41467-026-68337-z)

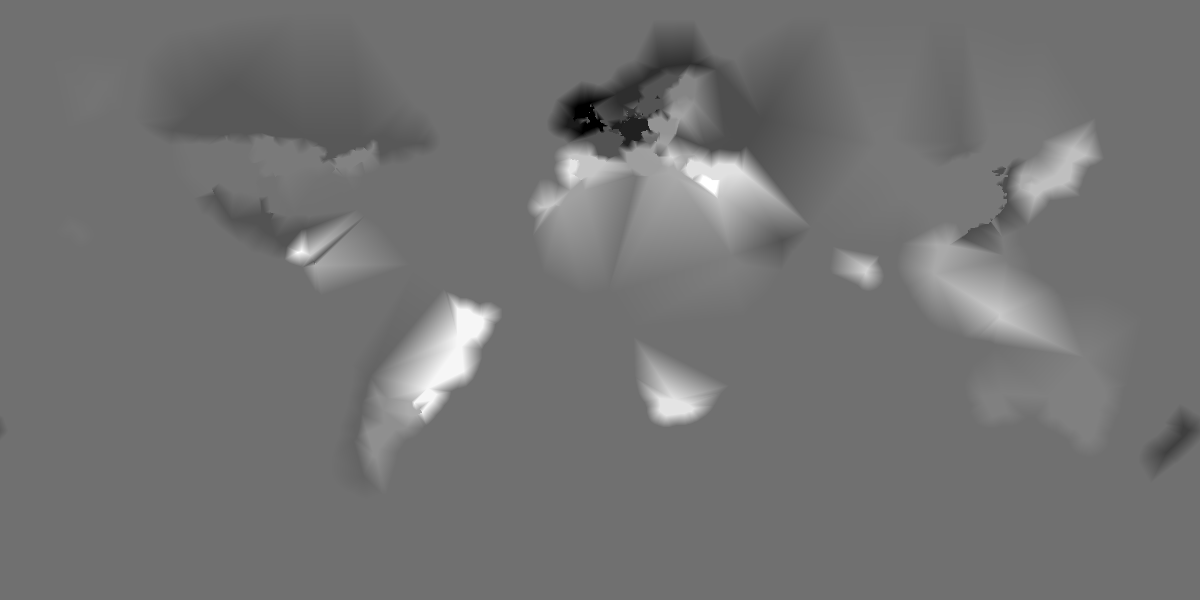

Supplement: Supplementary file 6 — Supplementary Data 4 [file 41467_2026_68337_MOESM6_ESM.tif]
